# Association between rectal gonorrhoea and HIV incidence in men who have sex with men: a meta-analysis

**DOI:** 10.1136/sextrans-2021-055254

**Published:** 2021-12-15

**Authors:** Deborah Donnell, Kidist Zewdie, Natasha Ratna, Veronica Miller, John Michael Saunders, O Noel Gill, Valerie Delpech, Hamish Mohammed

**Affiliations:** 1 Vaccine and Infectious Disease Division, Fred Hutchinson Cancer Research Center, Seattle, Washington, USA; 2 Department of Epidemiology, University of Washington, Seattle, Washington, USA; 3 Blood Safety, Hepatitis, Sexually Transmitted Infections (STI) and HIV Division—National Infection Service, Public Health England, London, UK; 4 School of Public Health, University of California System, Oakland, California, USA; 5 Forum for Collaborative Research, Washington, District of Columbia, USA; 6 HIV & STI Department, Public Health England, London, UK; 7 Research Department of Infection and Population Health, University College London, London, UK

**Keywords:** meta-analysis, HIV infections, *Neisseria gonorrhoeae*, men, public health

## Abstract

**Background:**

Incidence of rectal gonorrhoea (GC) has been hypothesised as a correlate of HIV exposure in prevention trials of men who have sex with men (MSM). High rectal GC incidence in MSM trials of new biomedical prevention drugs may provide supportive evidence for ongoing HIV risk. Empirical evidence of correlation between rectal GC and HIV incidence is needed to assess whether high rectal GC rates reliably correlate with high risk of HIV.

**Methods:**

Rectal GC and HIV are routinely tested in sexual health clinics (SHCs) throughout England. Through routine surveillance data collected at visits to SHCs, we assessed HIV incidence and new rectal GC diagnoses in repeat visits by HIV-negative MSM between 2011 and 2018, predating widespread roll-out of pre-exposure prophylaxis. Meta-analysis regression assessed population-level association between HIV and rectal GC incidence over time.

**Findings:**

Between 2011 and 2018, HIV and rectal GC incidence was assessed in 541 056 HIV-negative MSM attending SHCs in England. HIV incidence among MSM attending SHCs fell from 1.26/100 person-years (PYs) in 2011 to 0.28/100 PYs in 2018. Rectal GC rates increased from 3.5/100 PYs to 11.1/100 PYs over the same period. The rate of HIV incidence decreased by 22.3% for each percent increase in rectal GC (95% CI –30.8 to –14.7, p<0.001).

**Interpretation:**

Among the population of MSM attending SHCs in England, rectal GC rates increased substantially while HIV incidence rates decreased between 2011 and 2018. HIV incidence likely decreased through expanded HIV testing, prompt antiretroviral treatment (ART) initiation and increased viral suppression in persons living with HIV, interventions that did not decrease rectal GC. Rectal GC may not be an ideal proxy for HIV incidence in trials, as HIV exposure risk is complex and context dependent, given effective HIV prevention interventions in MSM.

Introduction

Measures that reliably predict risk of HIV have been sought and reported since the beginning of the HIV epidemic. The association between risk of incident rectal gonorrhoea (GC) infection and incident HIV infection in gay, bisexual and other men who have sex with men (MSM) is plausibly caused by a common route of exposure: condomless anal sex. Elevated risk of HIV associated with concurrent or prevalent rectal GC has been observed in MSM in multiple settings.^1–4^ A recent meta-regression analysis using eight studies published between 2000 and 2016 illustrated a strong positive ecological correlation between the incidence of HIV and rectal GC.^5^

Since daily tenofovir/emtricitabine (FTC/TDF) was established to be highly effective for HIV prevention in MSM, the field of HIV prevention has anticipated that rates of HIV in future trials could drop from ‘low’ (1%–2%) to extremely low, potentially below 0.5%. Recently, with the evidence of high effectiveness for both emtricitabine/tenofovir alafenamide (F/TAF) and injectable cabotegravir (CAB-LA),^6–8^ this future is now imminent. Effective antiretroviral (ARV)-based prevention of HIV has necessitated a change from superiority to non-inferiority hypotheses in prevention trial designs, and correspondingly, higher sample sizes.^9 10^ The combination of non-inferiority designs and extremely low rates of infection while using effective ARV-based prevention would require randomised clinical trial designs with expected trial sizes too large to be feasible, in part because the HIV epidemic remains concentrated in specific risk populations and regions. Consistency of effectiveness from four different classes and three different modalities of ARVs has also raised collective confidence in the prevention potential of ARV-based drugs.

We can now anticipate a future with near absence of observed HIV infections in clinical trials testing new ARV-based products: all persons in future clinical trials would receive an active ARV, and long-acting formulation has proven to be highly effective in preventing infections. The prospect of very low numbers of observed infections has led to serious scholastic attention on measures that could reliably predict or correlate with ongoing HIV exposure in a cohort enrolled in an HIV prevention trial. A correlate of HIV exposure would be a measure that (in the absence of effective biomedical prevention that prevents acquisition) reliably correlates with risk of HIV infection at the level of the population or cohort. If this measure accurately reflected ongoing HIV transmission risk in a cohort, even in the absence of HIV infections prevented by an effective intervention, we could infer that the ARV-based product is preventing HIV infection. A reliable measure of HIV exposure could potentially provide a line of supportive evidence for effectiveness of new ARV-based products, that is, estimate the population risk of HIV infection in the absence of the biomedical intervention.

In a future multisite trial with low observed number of HIV infections, inference about HIV risk based on a correlate of HIV exposure would be assessed by summarising rates over the entire cohort or within subgroups (eg, comparing rate of exposure across clinical sites or age subgroups). Thus, our focus in this study is whether group rates of HIV and rectal GC have strong positive correlation, that is, a high (low) rate of rectal GC in a cohort reliably indicates high (low) rates of HIV infection. We use a meta-regression approach to explore the association between rectal GC and HIV and assess the use of rectal GC as a proxy for HIV incidence, as a marker of ongoing HIV exposure in a cohort of HIV-negative MSM attending sexual health clinics (SHCs) in England from 2011 to 2018. Using data aggregated by calendar years and regions, we assess the population-level association between rectal GC and HIV.

## Methods

We used patient-level data from the GUMCAD STI surveillance system, a mandatory, pseudonymised dataset of all attendances in England at sexual health services (SHSs) which include SHCs providing specialist STI-related care as well as community-based settings commissioned to provide non-specialist STI-related care. Throughout the period of our assessment, the STI screening guidelines for MSM at high risk of STIs, established in 2014, were triple-site (pharyngeal, rectal and urethral) asymptomatic STI screening and HIV testing every 3 months.^11^ GUMCAD includes information on all STI and HIV testing, diagnoses and services provided alongside demographic characteristics for every patient attendance at SHSs in England starting 2009. Patient identifier codes are clinic specific, therefore visits occurring at different clinics cannot be linked in the dataset. Thus, the number of clients attending SHCs may count the same person more than once at different clinics. For this analysis, we extracted GUMCAD data on MSM (gay, bisexual and other men who ever reported having sex with men) at least 16 years of age, with SHC attendance between 2010 and 2019. Attendances were restricted to SHCs because testing, treatment and management of STIs among MSM are considered complex, and require specialist supervision.

Both incidence of rectal GC and HIV incidence were assessed for MSM who were initially HIV negative, corresponding to the population of interest for HIV prevention studies (ie, future HIV prevention studies will enrol a cohort of HIV-negative MSM). The criteria for inclusion in the analysis were report of sex with another man, age >16 years, HIV negative at the initial clinic visit and (for potential inclusion in incidence calculations) a repeat clinic visit. We excluded MSM who were on pre-exposure prophylaxis (PrEP) at the time of clinic attendance, to study the association between rectal GC and HIV in the absence of PrEP use.

Assessment of each of HIV incidence and rectal GC incidence uses distinct time periods for each calendar year. HIV incidence in a given calendar year was assessed by including all HIV tests from the same patient that include time in that year (provided the time between tests was less than 2 years). Only person time within the calendar year was included in the calculation. For HIV seroconverters, the time of seroconversion is imputed at the midpoint between the last HIV-negative and first HIV-positive test; the event is included if that date falls in the calendar year. Online supplemental figure 1 illustrates the computation of person-years (PYs) and the categorisation of infections by calendar year. Note that tests in the prior and subsequent year are used in the calculation of HIV incidence—that is, testing in 2009 and testing in 2011 are needed to assess incidence for 2010. HIV incidence for each calendar year is then calculated by aggregating the number of seroconverters observed in each year and dividing by the total number of PYs accumulated. Rectal GC incidence is assessed similarly, also restricted to initially HIV-negative MSM, except the time of the event was estimated as the time of the test. To avoid double-counting of repeat testing to confirm effective treatment, only one test or diagnosis of rectal GC is counted within a 42-day episode of care, otherwise distinct rectal GC diagnoses are assumed to be different incident infections. Multiple GC infections within the same year are included as distinct events. Rectal GC rates are calculated for each calendar year by aggregating the total number of infections observed in each year divided by the number of PYs accumulated.

10.1136/sextrans-2021-055254.supp1 Supplementary data

## Statistical methods

Reliable surrogates are established through consistency of association across multiple independent settings.^12^ In this study, we mimic sampling from independent settings within England by aggregating data on STI and HIV incidence within distinct time periods and from distinct geographical settings. A meta-regression for these observed aggregated data is used to study the association between HIV and STI incidence under the assumption of independence. Independence is justified because observations are distinct in time (events in 1 year are not included in another year) and by region. Data are independent geographically because inclusion requires longitudinal records within the same clinic, thus the number of patients included in more than one region’s data is likely to be small. Individual clients’ records cannot be linked across clinics in GUMCAD, thus a longitudinal record exists only within a given clinic.

We used random-effects meta-regression to model the linear association between (log) rate of HIV infection and rectal GC incidence, and assess the predicted rate increase in HIV incidence associated with each percent increase in rectal GC. For aggregated calendar year data, rectal GC incidence was the only model term; for calendar year by region, each region was fitted allowing separate intercepts and separate slopes per region. All statistical analyses were conducted using STATA V.15 (StataCorp, College Station, Texas, USA) and R V.4.0 (Vienna, Austria). Meta-regression modelling was performed in R (V.3.6) using the metafor R package.

We used Cox proportional hazards regression to assess the increase in individual-level probability of a new HIV infection diagnosis for those simultaneously diagnosed with rectal GC compared with those without rectal GC.

## Results

Of the 625 273 MSM identified in GUMCAD between 2010 and 2019, 541 056 had repeated clinic visits as the starting cohort for either HIV incidence or GC incidence calculation. The majority were white (78%), and 56% between the ages of 25 and 44 years old (table 1). Half (50%) of the MSM attendances occurred in London, with substantial patient volume (>55 000) also in northwest and southeast regions of England. The number of visits with STI testing increased steadily over the 9 years from approximately 200 000 in 2011 to 350 000 in 2018.

**Table 1:** Demographic characteristics of HIV-negative MSM attending sexual health clinics (2011–2018)

Characteristic	N (%)
**Age group (years**)	
16–24	134 544 (25)
25–34	193 128 (36)
35–44	110 381 (20)
45+	102 648 (19)
**Race/ethnicity**	
White	440 648 (78)
Black	16 619 (3)
Other	68 224 (13)
Unknown	35 565 (7)
**Residence**	
Not London/unknown	298 350 (55)
London	242 706 (45)
**Region**	
London	270 996 (50)
West Midlands	34 849 (6)
East of England	27 199 (5)
East Midlands	19 283 (3.6)
Northeast	14 435 (2.7)
Yorkshire and Humber	28 863 (5.3)
Northwest	58 952 (10.9)
Southwest	30 534 (5.6)
Southeast	55 945 (10.3)

MSM, men who have sex with men.

For the HIV incidence calculation for years 2011–2018, we included only those who had repeat visits with an HIV test within 6 months of the start and end of the year, which included 230 132 men. HIV incidence gradually declined from 1.26/100 PYs in 2011 to 0.94/100 PYs in 2015; in 2016, HIV incidence dropped to 0.56/100 PYs and steadily declined since, to a low of 0.28/100 PYs in 2018 (table 2).

**Table 2:** Number of HIV and rectal GC infections diagnosed and rates of infections by year

Year	Number of eligible HIV-uninfected MSM*	Number contributing to HIV incidence†	HIV diagnoses	Person-year	HIV incidence (95% CI)	Rectal GC diagnoses	Person-year	Rectal GC incidence (95% CI)
2011	90 264	40 164	326	25 923	1.26 (1.13 to 1.40)	487	14 125	3.45 (3.16 to 3.77)
2012	104 343	49 212	340	30 444	1.12 (1.00 to 1.24)	690	15 582	4.43 (4.11 to 4.77)
2013	111 978	55 804	415	36 046	1.15 (1.05 to 1.27)	1108	19 451	5.70 (5.37 to 6.04)
2014	128 136	64 490	446	41 038	1.09 (0.99 to 1.19)	1449	24 593	5.89 (5.60 to 6.20)
2015	138 382	71 253	429	45 528	0.94 (0.86 to 1.04)	1603	29 318	5.47 (5.20 to 5.74)
2016	141 588	74 891	276	49 002	0.56 (0.50 to 0.63)	1976	31 620	6.25 (5.98 to 6.53)
2017	151 295	80 710	187	52 579	0.36 (0.31 to 0.41)	2839	34 914	8.13 (7.84 to 8.44)
2018	152 319	82 082	153	55 343	0.28 (0.24 to 0.32)	4439	39 875	11.13 (10.81 to 11.46)

*MSM were eligible for inclusion if they were HIV uninfected and had two HIV test results.

†Person time from an MSM was included in calculation of HIV incidence for that calendar year if the interval between HIV test was contained within 6 months before (after) the beginning (end) of the calendar year.

GC, gonorrhoea; MSM, men who have sex with men.

Rectal GC incidence more than doubled between 2011 and 2017, from 3.45/100 PYs (487 diagnoses in 14 125 PYs) in 2011 to 8.13/100 PYs (2839 diagnoses in 34 914 PYs), reaching a new high of 11.13/100 PYs in 2018.

**Figure 1:**
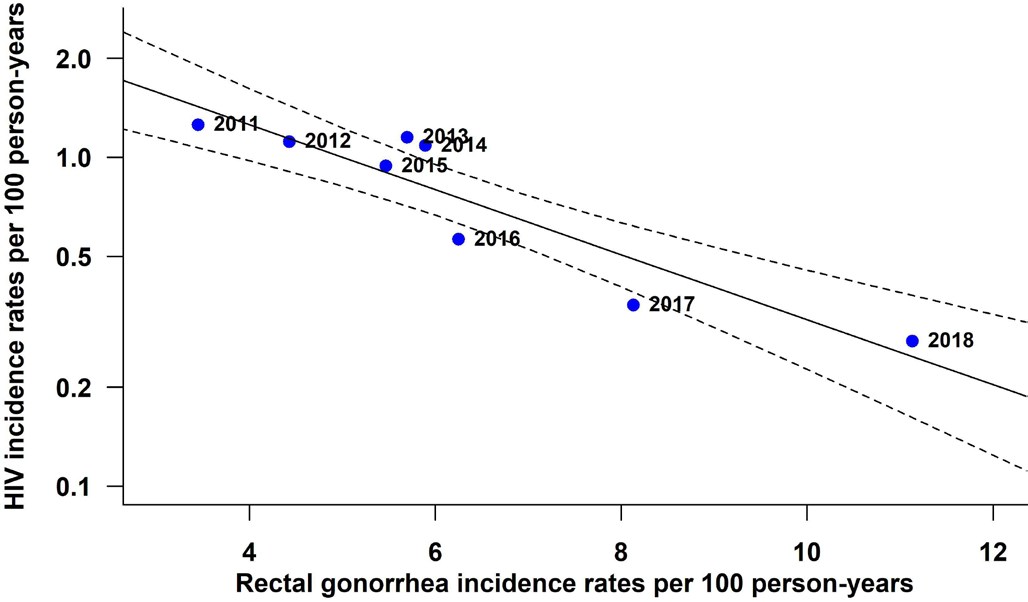
Association between annual incidence of HIV and annual incidence of rectal gonorrhoea for MSM attending sexual health clinics in England, 2011–2018. Fitted line and 95% confidence region for linear model from random-effects meta-regression of aggregate data for each year. MSM, men who have sex with men.

**Figure 2:**
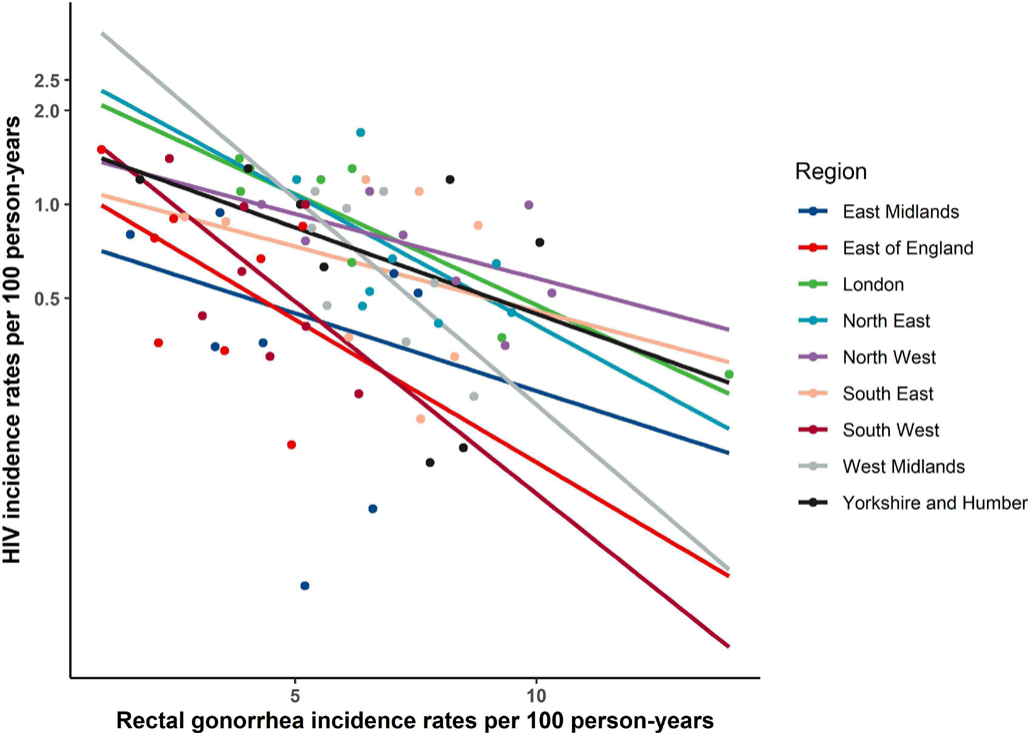
Association between annual log incidence of HIV and annual incidence of rectal gonorrhoea within public health regions of England for MSM attending sexual health clinics, 2011–2018. Fitted line is based on linear model from random-effects meta-regression of aggregate data for each year allowing distinct intercepts and slopes for each region. MSM, men who have sex with men.

Increasing rectal GC rates were associated with decreasing HIV incidence: the meta-regression estimate of annual (log) HIV incidence predicted by annual rectal GC incidence showed a slope of −0.223 (95% CI −0.308 to –0.147), or a 22.3% rate of decrease in HIV incidence associated with each percent increase in rectal GC (p<0.0001, figure 1). The models fitted to aggregate data across both calendar periods and regions showed a similar negative association in all regions (common slope=−0.149 (95% CI −0.204 to –0.093), p<0.0001): overall, the meta-regression showed a 14.9% rate of decrease in HIV incidence for every percent increase in rectal GC adjusted for region, with significant variation in HIV incidence between regions (figure 2).

In the individual-level analysis, the adjusted HR for a new diagnosis of HIV associated with a coincident diagnosis of rectal GC was 3.25 (95% CI 2.77 to 3.83), compared with MSM without a rectal GC infection. The unadjusted HR was 2.75 (95% CI 2.35 to 3.24).

## Discussion

A strong negative ecological association was observed between HIV and rectal GC incidence, the opposite of what was observed in a meta-analysis of trials conducted between 2000 and 2016, and the opposite of what would be epidemiologically expected for infections that share a common route of exposure. The association was quite consistent across different geographical regions, including settings with higher and lower incidences of HIV. Similar lack of ecological association between rectal STIs and HIV diagnoses has been found in other settings with MSM risk, also based on surveillance settings.^13 14^ However, a meta-analysis of rectal GC and HIV incidence across seven trials in varied settings showed a strong positive correlation.^5^ It is a common assumption in HIV prevention clinical trial settings that high prevalence of rectal STIs in a cohort of MSM is equated with evidence that the enrolled population is at high risk of HIV.^6 15^ Therefore, in a future trial of an HIV prevention product with unknown efficacy, researchers and regulators may be comfortable assuming that a clinical trial population is known to be at high risk of HIV because high rates of rectal GC were observed during follow-up of the cohort. Our data demonstrate the risk of an ecological fallacy in this assumption: in men attending SHCs in England, rectal GC rates continued to rise but risk of HIV infection did not.

Rectal GC remained a strong predictor of increased risk of HIV at the individual level, with three times the rate of HIV diagnosis among people presenting with a rectal GC infection than those without concomitant GC. This is consistent with similarly high association observed in MSM settings as diverse as Germany,^4^ Kenya^14^ and the USA.^1^ Note that this individual-level association is not an assessment of the population-level risk of HIV for a a given GC prevalence, that is, the probability of a population HIV incidence for a GC infection rate of 5/100 PYs versus a GC incidence of 10/100 PYs. In an illustration of ecological fallacy, while the individual association of HIV with rectal GC diagnosis is positive, at the population level, HIV incidence is declining during a period where rectal GC was steadily increasing.

A plausible explanation of our finding is the impact of increasing ARV treatment on HIV risk, that is, the strong prevention impact of decreased viral burden in England as a result of the change in ARV treatment guidelines^16 17^—an intervention that could have had a targeted impact on HIV incidence without changing the risk of rectal GC. Other prevention interventions ramping up during this time were increased STI/HIV testing.^18^ A substantial increase in SHC visit volume among HIV-uninfected MSM between 2010 and 2019 has been previously documented.^19^ This is the result of intensive efforts by sexual health and HIV professional bodies, Public Health England and sexual health charities to increase testing of both HIV and STIs over this 10-year period. Scale up of rapid turnaround tests at some large SHCs and the introduction of PrEP in 2017 also led to increases in STI/HIV testing.^20 21^ However, in SHCs, HIV and STI testing was routinely done at the same visit, thus impact of testing and treatment would likely have aligned effects on both infections. Changes in condom use would also have similar prevention effects on both infections. PrEP uptake in England before 2018 was modest but because of the potential to have an impact on HIV incidence, MSM using PrEP were excluded in our analysis. Others have suggested that increasing STIs in the population in a setting of decreasing or stable HIV is the result of harm reduction strategies such as sero-sorting or oral sex.^13 22 23^

This study highlights the difficulty of establishing a reliable surrogate of HIV exposure. A biological marker of an STI with the same route of exposure as HIV would appear to have strong potential to be a surrogate of HIV exposure. The strong and unexpected negative correlation demonstrates the challenges of characterising risk of infection from two different pathogens in an era where treatment has the potential to dramatically change infectiousness of one but not the other.

Our study demonstrates the possibility of epidemiological associations failing to be preserved across different settings, by population or time or place, as the relationship between HIV and rectal GC incidence may not remain constant. This compromises the use of rectal GC as a biological surrogate of HIV exposure in MSM, especially in settings where HIV incidence is falling as a result of successful prevention uptake.

**Key messages**

- HIV rates decreased while rectal gonorrhoea (GC) rates increased in men who have sex with men (MSM) in England between 2011 and 2018 when assessed at the broad community level.
- Individual-level risk of HIV to MSM was increased with new diagnosis of rectal GC, consistent with prior epidemiological studies.
- Rectal GC is not a reliable proxy for HIV exposure, likely because of effective interventions for decreasing HIV risk that do not affect risk of rectal GC.

## Data Availability

All data relevant to the study are included in the article or uploaded as supplemental information. Not applicable.
