# Supplementary material for: Association between rectal gonorrhoea and HIV incidence in men who have sex with men: a meta-analysis
Source: Sex Transm Infect. 2021 Dec 15;98(7):492–6. doi: 10.1136/sextrans-2021-055254 (PMC9613867; doi:10.1136/sextrans-2021-055254)

Supplementary Figure 1. Illustration of definition of person years and incident cases included in annual HIV incidence. Dots indicate HIV-negative tests, diamonds HIV-positive tests. Redlines are seroconverters (cases 1-4). Blue lines HIV-negative cases (Cases 5-10). Black arrows show the length of the person year intervals included. For seroconverters, the infection is assumed to occur at the midpoint, and only half the interval between tests is included. If the midpoint is not in the calendar year, the seroconverter is not included (Case 3). When the time between tests spans the year beginning/end, only time in the calendar year is included (Cases 2, 4-8), When the tests are more than two years apart, the data is excluded (case 10). HIV incidence rate is the number of observed seroconverters/sum of person years.

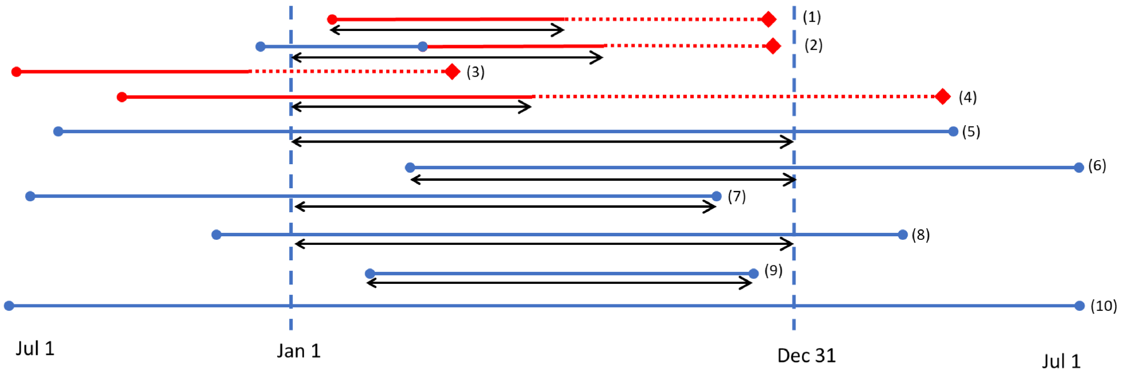

Supplement: Supplementary data [file sextrans-2021-055254supp001.pdf]
